# Pleuroperitoneal Fistula, an Unusual Case of Recurrent Unilateral Transudative Pleural Effusion

**DOI:** 10.1002/rcr2.70655

**Published:** 2026-06-25

**Authors:** Collin Kah Jing Lo, Yi‐De Ng, Grace Sook Hoon Low, Leng Cheng Sia

**Affiliations:** ^1^ Department of Respiratory Medicine Derriford Hospital, University Hospital Plymouth Trust Plymouth UK; ^2^ Department of Biomedical Imaging, Faculty of Medicine Universiti Malaya Research Imaging Centre (UMRIC), Universiti Malaya Kuala Lumpur Malaysia; ^3^ Internal Medicine Department Sunway Medical Centre Sunway City Malaysia; ^4^ Respiratory Department University Malaya Medical Centre (UMMC) Kuala Lumpur Malaysia; ^5^ Department of Medicine University of Malaya Kuala Lumpur Malaysia

**Keywords:** end stage renal failure, glucose gradient, peritoneal dialysis, pleural effusion, pleuroperitoneal fistulae

## Abstract

A 39‐year‐old woman afflicted by systemic lupus erythematous (SLE) with musculoskeletal involvement and end‐stage renal failure (ESRF) on continuous ambulatory peritoneal dialysis (CAPD) presented with recurrent left‐sided transudative pleural effusion. Guided by the ratio of pleural glucose to serum glucose > 1, and further confirmatory tests via a targeted imaging study, we were able to secure the diagnosis of a pleuroperitoneal leak. This case report highlighted the finding of this uncommon diagnosis that, if missed, would be detrimental to the patient's care.

## Graphical abstract

A 39‐year‐old woman afflicted by SLE with musculoskeletal involvement and ESRF on CAPD presented with recurrent left‐sided transudative pleural effusion. Guided by a glucose gradient of 2.6 mmol/L and further confirmatory tests via CT peritoneography, a diagnosis of a pleuroperitoneal leak was secured.

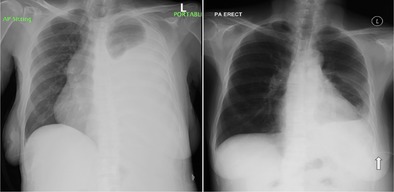

## 1 Introduction

Transudative pleural effusion in patients undergoing peritoneal dialysis is often treated as a sign of fluid overload, prompting optimisation of the dialysis regimen to better suit the patient's need. However, the occurrence of unilateral pleural effusion should warrant further investigation to determine its nature and aetiology, as transudative pleural effusions due to pleuroperitoneal communication can occur in 1.6%–10% of patients receiving peritoneal dialysis (PD) [1]. Here we would like to present a case of pleuroperitoneal fistulae in a woman who suffered from recurrent unilateral pleural effusion, of which the diagnosis was suspected based on glucose gradient difference and further confirmed on targeted imaging study.

## 2 Case Report

A 39‐year‐old woman with a background history of systemic lupus erythematous (SLE) (musculoskeletal and renal involvement), end‐stage renal failure (ESRF) on continuous ambulatory peritoneal dialysis (CAPD) for 4 years, presented to Emergency Department UMMC with increasing shortness of breath. Initial plain film chest X‐Ray revealed a massive left pleural effusion (Figure 1). A therapeutic aspiration was performed, which revealed a transudative profile with no positive growth in cultures and negative cytology. Reviewing her history of recurrent left‐sided pleural effusions, 2 therapeutic aspirations in previous admissions revealed similar findings (Table 1). In order to further investigate the possible aetiology of pleural fluid, pleural fluid and serum glucose levels were measured, demonstrating a pleural glucose level of 8.2 mmol/L and serum glucose of 5.6 mmol/L, corresponding to a gradient of 2.6 mmol/L.

**FIGURE 1:**
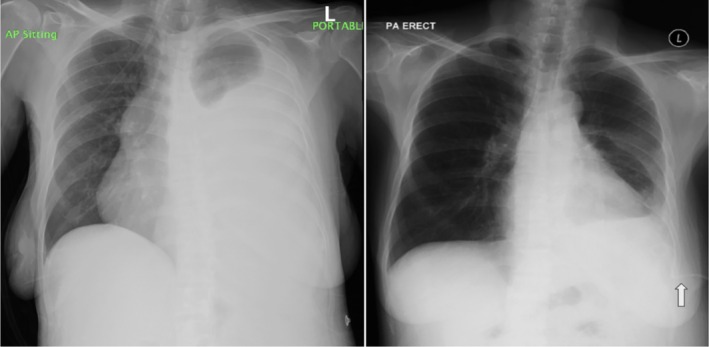
(Left) Massive left pleural effusion with deviated trachea and hear shadow to the right consistent with mass effect over the mediastinum. (Right) Post pigtail drainage (white arrow) demonstrated significant improvement of left pleural effusion with resolved mass effect onto mediastinum.

**TABLE 1:** Pleural and serum fluid laboratory results.

	Latest pleural fluid	Latest serum	Previous pleural fluid 1	Previous pleural fluid 2
Protein (g/L)	< 20	66	< 20	< 20
Lactose dehydrogenase (U/L)	72	292 (normal: 121–246)	40.14	44.97
Glucose (mmol/L)	8.2	5.6	NA	NA
Adenosine deaminase (U/L)	8.8 (< 40)	NR	NA	NA
White blood cell (U/L)	18	NR	Negative	68
Red blood cell (U/L)	Negative	NR	640	88
pH	8.0	NR	8.0	8.0
Cytology	Negative for malignant cells	NR	Small mature lymphocytes mixed with some mesothelial cells, degenerate cells, and occasional foam macrophages, no granuloma is detected. Negative for malignant cells.	Negative for malignant cells
Microbiology (fungal, bacterial, and mycobacterial cultures)	Negative	NR	Negative	Negative
Classification	Transudative	NR	Transudative	Transudative

Abbreviations: NA = not available; NR = not relevant.

CT peritoneography was performed by injecting 100 mL iopamiro 300 mg I/mL mixed in 2 L of dialysate and infused via Tenckhoff catheter (Figure 2). The patient was then instructed to lie supine for 30 min followed by 30 min left decubitus. The leak was demonstrated successfully in the left diaphragmatic dome, consistent with a pleuroperitoneal fistula. Following this, peritoneal dialysis was terminated and she underwent conventional haemodialysis for 6 months before receiving a living‐donor renal transplant. The pleural effusion was drained following the imaging study and remained minimal during 6 months of conventional dialysis before a complete resolution was achieved after receiving renal transplant.

**FIGURE 2:**
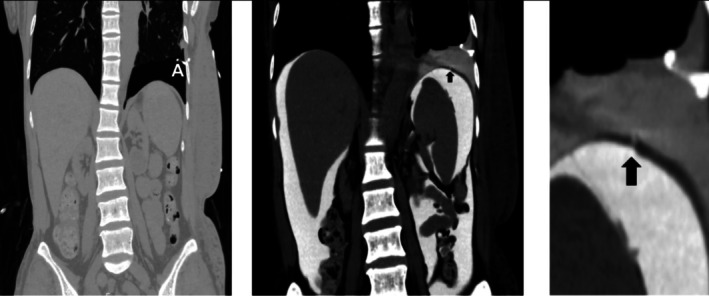
(Left) Conventional CT imaging demonstrates left pleural drainage catheter (A) in situ with no demonstrable pleuroperitoneal fistula. (Middle) CT peritoneography produces a diagnostic image demonstrating 2 mm left pleuroperitoneal fistula (black arrow). Peritoneal fluid crosses via fistula and accumulates in the chest cavity. (Right) Magnified image demonstrating the presence of small pleuroperitoneal fistula (black arrow).

## 3 Discussion

Pleuroperitoneal fistulae are a rare complication of CAPD. Patients usually present with breathlessness following initiation of peritoneal dialysis and demonstrate findings of pleural effusion. It is important to recognise that there is no specific time frame for pleuroperitoneal fistula to develop; it can form within weeks, months or even years. Our patient had undergone peritoneal dialysis for 4 years to date of presentation.

Two modalities of investigations involving pleural fluid analysis and imaging studies are considered to be helpful in making the diagnosis of pleuroperitoneal leak. A pleural fluid analysis which demonstrates transudative pleural fluid and high pleural glucose level can be suggestive of a potential pleuroperitoneal leak, whereas the demonstration of pleuroperitoneal leak via imaging studies can be diagnostic.

Although there are many imaging and laboratory investigation modalities being reported in the literature, the absence of consensus criterion creates a diagnostic dilemma for clinicians. Pleural glucose and serum glucose gradient of 50 mg/dL (2.8 mmol/L) was reported to have 100% specificity [2], however, it lacks in power as the study included only 2 patients. In cases in which serum glucose level is < 50 mg/dL (2.8 mmol/L), a pleural glucose to serum glucose ratio > 1 can be used to indicate the diagnosis [3].

Imaging modality such as CT peritoneography has lower sensitivity but demonstrates high specificity when fistulae are detected. It is noted that an increased pressure gradient between peritoneal cavity and pleural cavity can improve the sensitivity of imaging studies. It was reported that the CT peritoneography did not demonstrate the leakage until the patient inhaled maximally and strained to increase the pressure gradient [4].

In patients who are unable to discontinue peritoneal dialysis, other measures both conservative and invasive approaches can be considered. Conservative measurements such as peritoneal dialysis with low exchange in a semi upright position can be attempted, though recurrence is high. If that fails, more invasive approaches, including chemical pleurodesis and surgical correction, for example, surgical pleurectomy, mechanical abrasion and pleurodesis, and diaphragmatic patching via open surgery or video‐assisted surgery, can be considered [5].

In our case, following the gradient difference of 2.6 mol/L, we utilised the pleural glucose to serum glucose ratio > 1 and prompted the CT peritoneography. We were able to demonstrate a finding of pleuroperitoneal leak with a gradient of 2.6 mmol/L suggesting that a lower gradient such as 2.6 mmol/L may be helpful to screen, and the diagnosis can be further confirmed by imaging protocol, with emphasis on achieving a pressure gradient.

Pleuroperitoneal fistulae are a rare but important complication of peritoneal dialysis. If missed, transudative pleural effusions can be interpreted as inadequate optimisation of dialysis regimen or fluid overload from other aetiology. Diagnostic pleural fluid aspiration to differentiate a transudative versus exudative cause, followed by specifically tailored tests such as pleuro‐peritoneal glucose gradient level, should be performed. This could direct the targeted CT imaging in patients undergoing peritoneal dialysis with a suspicion of pleuroperitoneal fistula. This diagnosis is vital as its management includes the immediate cessation of peritoneal dialysis and consideration of other modes of renal replacement therapies. Currently, with the lack of available gold standard diagnostic criteria, we suggest utilising a gradient of 2.6 mmol/L to assist in the initial investigation and further confirmation by imaging protocol that puts emphasis on achieving a pressure gradient.

## Author Contributions

Yi‐De Ng – Radiologist who assisted in carrying out the diagnostic imaging study and confirmed the diagnosis. Grace Sook Hoon Low – Consultant general medicine who is responsible for providing patient care. Leng Cheng Sia – Supervisor, corresponding author and Consultant Respiratory Medicine who is responsible for providing patient care. Collin Kah Jing Lo was a student of Dr. Leng Cheng Sia previously and has now moved to the United Kingdom for further training at the time of this writing.

## Funding

The authors have nothing to report.

## Consent

The authors declare that written informed consent was obtained for the publication of this manuscript and accompanying images using the consent form provided by the journal.

## Conflicts of Interest

The authors declare no conflicts of interest.

## Data Availability

The data that support the findings of this study are available from the corresponding author upon reasonable request.
